# Three-dimensional dynamics optical coherence tomography for tumor spheroid evaluation

**DOI:** 10.1364/BOE.440444

**Published:** 2021-10-12

**Authors:** Ibrahim Abd El-Sadek, Arata Miyazawa, Larina Tzu-Wei Shen, Shuichi Makita, Pradipta Mukherjee, Antonia Lichtenegger, Satoshi Matsusaka, Yoshiaki Yasuno

**Affiliations:** 1 Computational Optics Group, University of Tsukuba, Tsukuba, Ibaraki 305-8573, Japan; 2 Department of Physics, Faculty of Science, Damietta University, New Damietta City, 34517, Damietta, Egypt; 3 Sky Technology Inc., Tsukuba, Ibaraki 305-0032, Japan; 4 Clinical Research and Regional Innovation, Faculty of Medicine, University of Tsukuba, Ibaraki 305-8575, Japan; 5 Center for Medical Physics and Biomedical Engineering, Medical University of Vienna, Währinger Gürtel 18-20, 4L, 1090, Vienna, Austria

## Abstract

We present a completely label-free three-dimensional (3D) optical coherence tomography (OCT)-based tissue dynamics imaging method for visualization and quantification of the metabolic and necrotic activities of tumor spheroid. Our method is based on a custom 3D scanning protocol that is designed to capture volumetric tissue dynamics tomography images only in a few tens of seconds. The method was applied to the evaluation of a tumor spheroid. The time-course viability alteration and anti-cancer drug response of the spheroid were visualized qualitatively and analyzed quantitatively. The similarity between the OCT-based dynamics images and fluorescence microscope images was also demonstrated.

## 1. Introduction

Recently, precision medicine-based disease treatment has attracted the attention of medical researchers. This protocol takes the genes, the environment, and the life style variability of each patient into account to enable accurate therapy for specific diseases [1].

Cancer is a disease with one of the highest fatality rates worldwide [2]. Cancer can be caused by abnormal proliferation and mutation of several cell types, which mean that there are hundreds of types of cancer that depend on the type of cell that becomes cancerous. As a result, cancer types vary from patient to patient [3]. Survival rates from cancer can be increased by early diagnosis and optimal selection of anti-cancer drugs for individual patients. Therefore, precision medicine could be useful for cancer treatment.

One of the most important precision medicine strategies for cancer treatment is to mimic the living tissues by *ex vivo* culturing of patient-derived cancer cells to form a three-dimensional (3D) multi-cellular tissue called a patient-specific tumor spheroid [4,5]. These tumor spheroids emulate *in vivo* solid tumors more closely than 2D monolayer tumor models, because of their heterogeneous structures, growth kinetics, and cell interactions. Therefore, these spheroids are useful for gaining a deeper understanding of the tumor biology, and are also useful for the anti-cancer drug investigations by measuring its responses to particular drugs [6–8].

Assessments of tumor spheroids can be performed by several conventional methods. Staining histology is one conventional imaging modality used for tumor spheroid evaluation [9–11]. However, this method is destructive because it requires the tissue to be sliced, which may damage the tissue’s micro structures and even alter the functionality of the tumor cells. In addition, this histology imaging method only provides 2D images, which are not sufficient for the evaluation of 3D spheroids.

Fluorescence microscopy is a gold standard modality for tumor spheroid tissue viability and tumor spheroid-based drug response evaluation [7,12–14]. However, fluorescence imaging requires the use of fluorophores as markers, which will then disturb the cellular environment. In addition, this method may suffer from photo bleaching, which will reduce the quantitative assessment capability of the method [15,16]. Furthermore, deep tissue imaging cannot be performed by fluorescence microscopy because of its limited imaging penetration depth.

Time-course tracking of alterations in tissue morphology through microscopic imaging processes has been used for tumor spheroid-based anti-cancer drug selection [17,18]. These methods are based on use of a bright field microscope and a fluorescence microscope, which means that the tissue specificity is weak and the method is not really quantitative. Because of these limitations, this method requires several days or even weeks to select the appropriate drugs. This long selection time may thus have a negative effect on the cancer survival rate.

The limitations of the conventional methods described above are summarized as follows. First, these methods are invasive, i.e., we cannot assess the cells in their real natural condition. Second, these methods do not have a volumetric imaging capability. These limitations can be partially overcome by using optical coherence tomography (OCT). OCT is a nondestructive, label-free, high-resolution, and volumetric imaging modality with an imaging depth of a few millimeters [19]. A microscopic version of OCT called optical coherence microscopy (OCM) has been used previously to perform cellular-scale imaging [20–22].

However, conventional OCM can only visualize the morphology of the samples. Therefore, OCM is also not suitable for the spheroid evaluation. To enable use of OCM as a spheroid evaluation tool, it is necessary to extend the contrasts of OCM. Polarization-sensitive OCT [23–28] has been shown to give specific contrasts for collagen and melanin. Attenuation coefficient (AC) imaging has proved to be sensitive to tissue density [29–31]. In addition, optical coherence elastography [32–34] can assess the mechanical properties of samples.

Among the existing contrast extensions for OCT, tissue dynamics imaging is an recently emerging modality. This method visualizes and quantifies the sub-cellular motion of the tissue, which is closely associated with both cell function and cell viability. *En face* dynamics OCT has been demonstrated to enable visualization of the tissue metabolism by combining time-domain full-field OCT with time-spectroscopic analysis [35–38]. Cross-sectional dynamics imaging has also been performed [39,40] by combining scanning OCT with time-spectroscopic analysis. We have recently demonstrated tumor spheroid evaluation via the temporal variance and decorrelation of the OCT signal intensity [41,42]. Although these methods are useful for the assessment of tissue and cell functions, they are all 2D imaging methods and thus can not be used for volumetric evaluation of the spheroid. There have been some demonstrations of 3D dynamics imaging [39,43], but these modalities requires long measurement times [39] or the use of an elaborate ultra-fast OCT system [43].

In this paper, we demonstrate 3D dynamics OCT imaging of tumor spheroids. The volumetric dynamics imaging is achieved using the combination of a standard speed (50,000 A-lines/s) OCT system and a newly developed scanning protocol. The utility of our method has been investigated via two types of study that involved human breast cancer cell spheroids. The first is a time-course longitudinal volumetric evaluation of the spheroids. The second is the evaluation of spheroid viability under anti-cancer drug application. These evaluation consists of not only the volumetric visualization but also quantitative analysis of the entire spheroid volume.

## 2. Principle: 3D dynamics OCT imaging

### 2.1 OCT device

A polarization-sensitive Jones matrix swept-source OCT system is used to perform the 3D tissue dynamics imaging [44,45]. The central wavelength of this system is 1.3 μ m and its scanning speed is 50 kHz. Our system provides an axial resolution of 14 μ m in tissue, while its lateral resolution (1/ $e^{2}$ -width) is 19 μ m. The lateral and axial pixel separations are 1.95 μ m and 7.24 μ m, respectively. The complete system specification has been published previously elsewhere [42,44]. Although this system is polarization sensitive, its polarization imaging capability is not used in this study. Only the average intensity of four OCT images from the system’s four polarization channels is used to perform the dynamics imaging.

### 2.2 Scan protocol for 3D dynamics OCT imaging

**Fig. 1.**
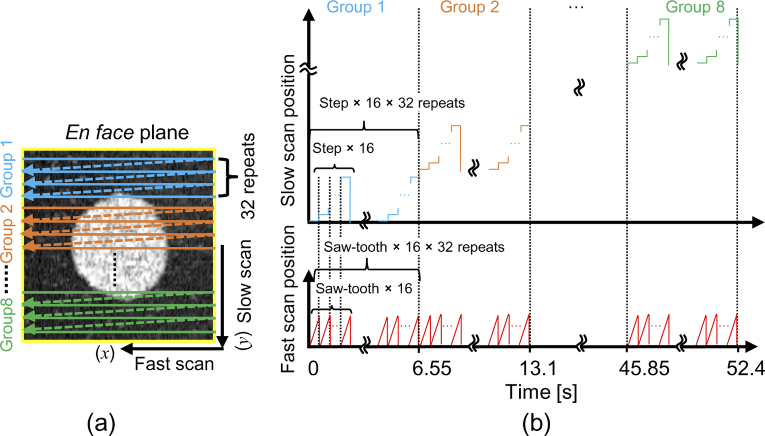
Schematic diagram of the 3D dynamics scanning protocol. (a) The protocol repeats a raster scan 32 times over the area of each group. (b) Time chart for the scan pattern.

Figure 1 shows a schematic of the scan protocol used for 3D tissue dynamics imaging. The basic concept of our protocol involves splitting the *en face* plane into eight groups, where each group consists of 16 locations in the tissue, as illustrated in Fig. 1(a). The first group is scanned by raster scanning and this procedure is repeated for 32 times in 6.55 s as shown in the time chart [Fig. 1(b)]. So, each location in the first group is scanned 32 times in 6.55 s (6.554 s to be exact), which leads to a B-scan repetition time of 204.8 ms. Then the slow scanning mirror moves to the next group and the same scanning pattern is then used to scan this group. The same process is repeated for the other groups. Finally, a volume composed of 128 locations in the tissue is captured in 52.4 s. The scanning area has dimensions of 1 × 1 ${\mathrm{mm}}^{2}$ , the total number of B-scans per unit volume is 4,096 B-scans, and each B-scan consists of 512 A-lines.

### 2.3 Signal processing for tissue dynamics imaging

To evaluate the 3D tissue dynamics, we used our previously demonstrated OCT time-sequence signal analysis methods, which are described below.

#### 2.3.1 Logarithmic intensity variance (LIV)

The logarithmic intensity variance (LIV) is the time variance of the log (-dB)-scale OCT time sequence signal at each pixel in the image.

(1) $$LIV(x,z)=\frac{1}{N}\sum_{i=0}^{N-1}{\left[I_{dB}(x,z,t_{i})-{\left\langle I_{dB}(x,z)\right\rangle }_{t}\right]}^{2}\;,$$

where $t_{i}$ is the sampling time of the i -th frame, where i = 0, 1, 2,…., N -1, and N is the total number of frames used to perform the computation. ${\left\langle \right\rangle }_{t}$ represents the average of the repeated B-scans intensities over the acquisition time t at the same location within the tissue. The LIV is sensitive to the magnitude of the OCT signal fluctuations. Further details about the LIV have been published elsewhere [42]. The LIV volume of 128 B-scans performed at 128 different locations in the tissue is obtained by computing Eq. (1) at each location using the sequentially captured B-scans ( N = 32) that were acquired using the 3D protocol described in Section 2.2.

#### 2.3.2 Late OCT correlation decay speed ( ${\mathrm{OCDS}}_{l}$ )

**Fig. 2.**
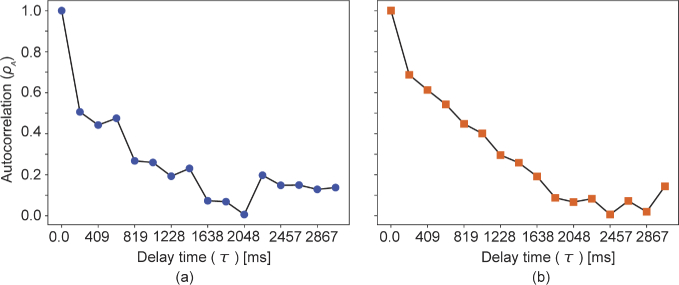
Examples of the autocorrelation decay curves. (a) shows the correlation when computed with a spatially non-extended (single pixel) kernel, while the curve in (b) is computed with the 2 × 4 pixels kernel.

The late OCT correlation decay speed ( ${\mathrm{OCDS}}_{l}$ ) is the slope of the autocorrelation decay curve at late delay times. ${\mathrm{OCDS}}_{l}$ is sensitive to the slow tissue dynamics and the correlation decay analysis method is described in detail in our previous publication [42]. In that previous publication, the autocorrelation function was computed at each pixel using 350 frames, which meant that each autocorrelation function is computed from 350 data points. In contrast, the 3D acquisition protocol proposed in this paper can compute the autocorrelation function from only 32 frames. The use of this small number of data results in a noisy autocorrelation decay curve, as exemplified by the curve shown in Fig. 2(a), and thus results in a noisy ${\mathrm{OCDS}}_{l}$ image. To overcome this noisy decay profile, we have revised the computation of the autocorrelation function by using a small spatial kernel, which results in a smooth decay curve, as shown in Fig. 2(b).

The autocorrelation function of the OCT time sequential signal is computed as follows

(2) $$\rho_{A}(\tau_{i};x,z)=\frac{Cov\left[I_{dB}(x,z,t_{i}),\;I_{dB}(x,z,t_{i}+\tau_{i})\right]}{Var\left[I_{dB}(x,z,t_{i})\right]Var\left[I_{dB}(x,z,t_{i}+\tau_{i})\right]},$$

where the numerator represents the covariance between $I_{dB}(x,z,t_{i})$ and $I_{dB}(x,z,t_{i}+\tau_{i})$ , and Var[] represents the variance. $\tau_{i}$ is the delay time defined as iΔt , where i is an integer variable. Δt is the B-scan repetition time, which is 204.8 ms in this case.

By substituting in the covariance and the variances and computing them over a kernel with a size of ( k×l ), Eq. (2) can then be written as

(3) $$\begin{array}{cc} \rho_{A}(\tau_{i};x,z) & =\frac{\frac{\sum_{k}\sum_{l}\sum_{t_{i}}^{\tau -\tau_{i}}[I_{dB}(x,z,t_{i})-\bar{I_{dB}(x,z,t_{i})}][I_{dB}(x,z,t_{i}+\tau_{i})-\bar{I_{dB}(x,z,t_{i}+\tau_{i})}]}{kl(\tau -\tau_{i})}}{\frac{\sum_{k}\sum_{l}\sum_{t_{i}}^{\tau -\tau_{i}}{\left[I_{dB}(x,z,t_{i})-\bar{I_{dB}(x,z,t_{i})}\right]}^{2}}{kl(\tau -\tau_{i})}\frac{\sum_{k}\sum_{l}\sum_{t_{i}}^{\tau -\tau_{i}}{\left[I_{dB}(x,z,t_{i}+\tau_{i})-\bar{I_{dB}(x,z,t_{i}+\tau_{i})}\right]}^{2}}{kl(\tau -\tau_{i})}}\\ & =\frac{\sum_{k}\sum_{l}\sum_{t_{i}}^{\tau -\tau_{i}}\left[I_{dB}(x,z,t_{i})-\bar{I_{dB}(x,z,t_{i})}][I_{dB}(x,z,t_{i}+\tau_{i})-\bar{I_{dB}(x,z,t_{i}+\tau_{i})}\right]}{\sum_{k}\sum_{l}\sum_{t_{i}}^{\tau -\tau_{i}}{\left[I_{dB}(x,z,t_{i})-\bar{I_{dB}(x,z,t_{i})}\right]}^{2}\sum_{k}\sum_{l}\sum_{t_{i}}^{\tau -\tau_{i}}{\left[I_{dB}(x,z,t_{i}+\tau_{i})-\bar{I_{dB}(x,z,t_{i}+\tau_{i})}\right]}^{2}}, \end{array}$$

where τ is the maximum delay time.

Equation (3) was implemented using a fast Fourier transform-based method (see [46] and the Appendix of [47]). In our particular case, the spatial kernel size was 2 × 4 pixels in the axial and lateral directions, respectively. The ${\mathrm{OCDS}}_{l}$ is then computed as the slope of the autocorrelation curve over the delay range of $\tau_{i}$ = [204.8, 1228.8 ms].

The tissue dynamics images are displayed as hue, saturation, value (HSV) color maps in which the dynamics value (i.e., the LIV or ${\mathrm{OCDS}}_{l}$ ) is set as the hue channel, the OCT intensity represents the value channel, and the saturation is set as 1 for all the pixels.

### 2.4 Comparison between cross-sectional and volumetric dynamics imaging

In our previous cross-sectional dynamics imaging approach, the system captured 350 B-scans at the same location in the tissue in 4.48 s [42]. The data size and the data acquisition time were suitable for use in cross-sectional imaging, but use of the same protocol for 3D volumetric data acquisition, which requires scanning of more than 100 locations in the tissue, would require several minutes. This long acquisition time is not practical for use in real-world applications.

However, by performing the optimization analysis that was described in the same article (Section 5.3), it was found that acquisition of 17 or 33 frames per location would be sufficient to perform tissue dynamics imaging if the time separation between the first and last frames was sufficiently long, e.g., 6.55 s. This motivated us to formulate the 3D scan protocol used in this work, which allows volumetric dynamics imaging to be performed with acquisition of a significantly smaller number of frames for each location than in the previous method, as described in Section 2.2.

Table 1 summarizes the main points of comparison between our previous 2D cross-sectional dynamics imaging scan protocol [42], and the newly proposed volumetric scan protocol. As the table shows, the new volumetric scan protocol can investigate 128 locations within a realistic acquisition time (52.4 s), while the old protocol would take more than 10 times that acquisition time for the same task. The rapid acquisition capability of the new protocol thus enables its use in volumetric dynamics imaging.

**Table 1.** Comparison of cross-sectional and volumetric dynamics imaging methods.

	Old 2D protocol [42]	New volumetric protocol
Number of frames per location	350 B-scans	32 B-scans
Acquisition time for 128 locations	573.44 s	52.4 s
Volumetric quantification	Not realistic	Yes
Inter-frame time separation	12.8 ms	204.8 ms
Fastest measurable dynamics	39.06 Hz	2.44 Hz
Slow dynamics (hundreds of ms)	Measurable	Measurable
Fast dynamics (tens of ms)	Measurable	Not measurable

However, the fastest measurable dynamics signal of the new protocol is at a frequency of only 2.44 Hz, whereas it is at a frequency of 39.06 Hz with the old protocol. Therefore, the new protocol is not compatible with our previously demonstrated “early OCT correlation decay speed ( ${\mathrm{OCDS}}_{e}$ )” approach [42]. The development of a volumetric scan protocol that is compatible with ${\mathrm{OCDS}}_{e}$ will thus be a part of future work.

## 3. Validation studies: tumor spheroid 3D tissue activity quantification

### 3.1 Samples and protocols

To evaluate the proposed 3D tissue dynamics method, we have used human breast adenocarcinoma spheroids (MCF-7 cell line). Two studies were organized to complete this validation process.

The first study (Study 1) included time-course imaging of the MCF-7 tumor spheroid for up to 20 h. This study was organized to validate the longitudinal imaging capability of the proposed method. Human breast-derived tumor cells have been seeded in 96-well ultra-low attachment plate to form the tumor spheroid. 1,000 cells per well were cultured to form the 3D tumor spheroid. The cell culture medium contained Eagle’s minimal essential medium (EMEM)/F12 (1:1) (Invitrogen, Waltham, MA) with a 2% B-27 supplement (Invitrogen), 2 ng/mL of basic fibroblast growth factor (bFGF; Wako, Osaka, Japan), 2 ng/mL of epidermal growth factor (EGF; Sigma-Aldrich, St. Louis, MO), 100 U/mL of penicillin G, and 0.1 mg/mL of streptomycin sulfate (Wako, Osaka, Japan). After 15 days, a spheroid with a diameter of approximately 500 μ m is formed. On day 15, the spheroid was extracted from the cultivation environment, stored in the same culture medium, and 30 min after extraction, 3D OCT measurements were performed at a room temperature of ${\mathrm{25}}^{\circ }$ C every 4 h up to 20 h. Notably, during the spheroid cultivation process, the samples were supplied with ${\mathrm{CO}}_{2}$ , while there was no ${\mathrm{CO}}_{2}$ supply during OCT measurement. Particular protocols were not used to avoid the contamination during the measurement.

The second study (Study 2) represents the drug response evaluation of the MCF-7 spheroid. In this study, 500 tumor cells per well were seeded in six wells of 96 well plate. After four days, six spheroids were formed in six wells. The samples were divided into two groups, with each group containing three spheroids. One of the three spheroids from each group is kept as a control and the other two spheroids were treated with 0.1 μ M and 1 μ M of Paclitaxel (PTX, Taxol). The first group was incubated with the drug for 24 h and then measured using the fluorescence microscope described in Section 3.2, and then the 3-D OCT measurements were performed. The second group was incubated for 72 h and then measured in the same manner as the first group. Note that all the samples were seeded at the same time. This means that the control sample of the 72 h treatment group was cultured for 48 h longer than the control sample of the 24 h treatment group.

### 3.2 Fluorescence microscopic imaging

Fluorescence microscopic imaging was performed using a THUNDER imaging system (Leica Micro-systems, Wetzler, Germany). A microscopic objective with a numerical aperture (NA) of 0.12 was used. Two fluorophores were applied to the spheroids for 3 h before the fluorescence microscopic measurements were taken. The first fluorophore was calcein acetoxymethyl (calcein-AM), which stains the viable cells and emits a green fluorescence. The second fluorophore was propidium iodide (PI), which highlights the dead cells with a red fluorescence.

### 3.3 Volumetric quantification of the MCF-7 spheroid

To quantify the volumetric alterations in the spheroid tissue viability in both longitudinal and drug response studies, the spheroid volume was extracted using an OCT intensity-based mask. The spheroid region has been selected manually but roughly using a polygon shaped region of interest (ROI). Then, by applying an intensity threshold over this ROI, the intensity-based mask is obtained. This process was performed for all the B-scans and a volumetric mask was thus obtained. The spheroid volume was then computed from this volumetric mask. The mean values of the LIV and the ${\mathrm{OCDS}}_{l}$ through the entire spheroid volume were subsequently computed. By applying an empirically defined LIV and ${\mathrm{OCDS}}_{l}$ cut-offs of 3 ${\mathrm{dB}}^{2}$ and 2 $\times 10^{-4}$ ${\mathrm{ms}}^{-1}$ , respectively, the viable volume has been computed. Then the viable cell ratio is computed as the volume ratio of the viable region over the whole spheroid; viable cell ratio = viable volume / entire spheroid volume. Here the volumes were obtained by counting the number of pixels in the corresponding regions.

## 4. Results

### 4.1 Study 1: 3D time-course evaluation of MCF-7 spheroid

**Fig. 3.**
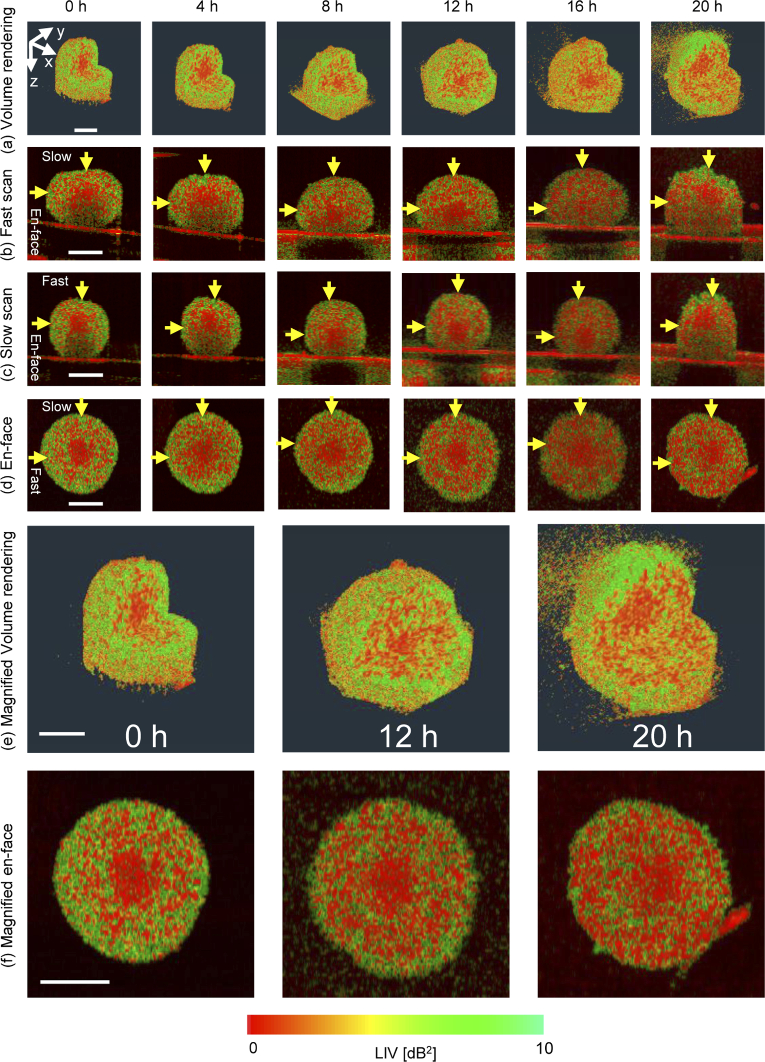
Time-course LIV visualization of MCF-7 spheroid formed by initial seeding of 1,000 tumor cells. The first to fourth rows show time-course images of (a) the cut-away volume rendering, (b) the cross-section along the fast scan direction, (c) the cross-section along the slow scan direction, and (d) the *en face* cross-section, respectively. The fifth and sixth rows show magnified images of (e) the volume rendering and (f) the *en face* cross-section, respectively. All the scale bars represent 200 μ m.

Figure 3 summarizes the time-course LIV imaging of the MCF-7 spheroid. The first to fourth rows represent the cut-away volume rendering, fast scan, slow scan, and *en face* LIV cross-section images, respectively. The fifth and sixth rows represent the magnified images of the volume rendering and the *en face* cross-sections, respectively, at 0, 12, and 20 h time points, receptively. The column index indicates the measurement time point for each image. It should be noted that the locations of the cross-sectional and the *en face* planes were manually selected at the spheroid center. However, it is not guaranteed that they are perfectly collocated among all the time points because of two reasons. At first, the spheroid was immersed in a liquid, so it might be slightly floating or rotating over time. The second, slight morphological alteration, such as increasing of the volume, occurred as discussed later in this section.

At the 0 h time point, the volume rendering and the cross-sectional views show that the spheroid core exhibited a low LIV signal. In contrast, the outer shell of the spheroid shows high LIV. The tumor spheroid is well known to have a necrotic core and proliferating cancer cells are located at the periphery of the spheroid [5,48]. The low LIV observed at the spheroid core may thus be related to the core necrosis, while the high LIV observed at the spheroid periphery may indicate the cell viability of the proliferating outer shell of the spheroid.

Time-course LIV shows that the size of the necrotic tissue region (with low LIV signal) increases gradually until it reaches almost the entire spheroid volume after 20 h. This reduction in the cell viability and increase in the size of the necrotic region may be caused by the lack of the nutrient supply, where the spheroid has been extracted from the cultivation environment and measured at room temperature without a ${\mathrm{CO}}_{2}$ supply over 20 h as noted in Section 3.1. It is noteworthy that the volume rendering images [Figs. 3(a) and 3(e)] show some scattering signals in the culture medium especially at 16 h and 20 h. It is because the culture medium became dirty and opaque at the late time points.

**Fig. 4.**
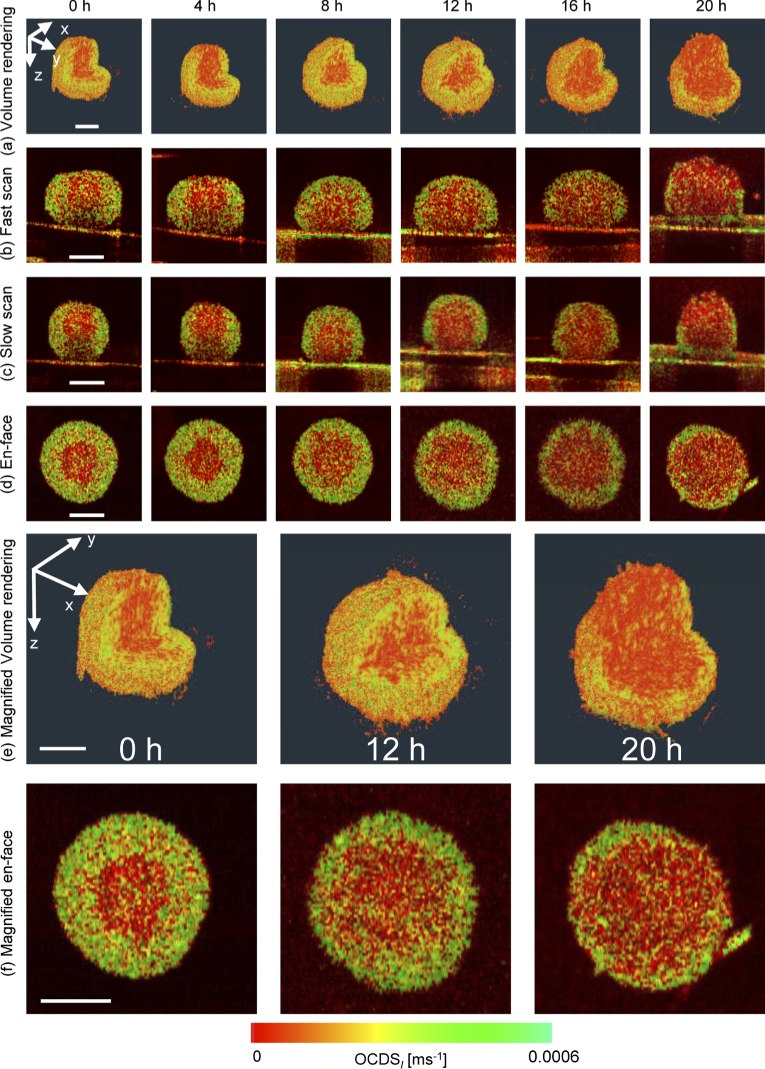
Time-course ${\mathrm{OCDS}}_{l}$ of MCF-7 spheroid formed by initially seeding of 1,000 tumor cells. The images are displayed in the same manner as those shown in Fig. 3.

Figure 4 shows the time-course ${\mathrm{OCDS}}_{l}$ images of the MCF-7 spheroid. Tendencies similar to those shown in the LIV images are obtained. The low ${\mathrm{OCDS}}_{l}$ at the spheroid core at the 0 h time point may be related to low tissue activity at the core region, which may in turn be related to the tissue’s necrotic cell death. In contrast, the high ${\mathrm{OCDS}}_{l}$ observed at the outer shell may be related to the high metabolic activity that occurs at this layer of viable cells. The time-course ${\mathrm{OCDS}}_{l}$ results shows a clear thinning of the high ${\mathrm{OCDS}}_{l}$ outer shell. This thinning may indicate degradation of the cell viability and an increase in tissue necrosis. The low ${\mathrm{OCDS}}_{l}$ volume increases to cover almost all the entire spheroid volume after 20 h, as shown in the magnified ${\mathrm{OCDS}}_{l}$ volume rendering and *en face* images [Figs. 4(e) and 4(f), respectively].

**Fig. 5.**
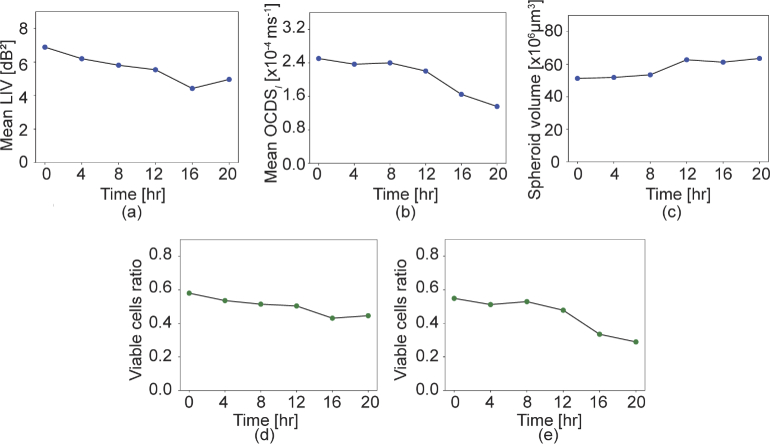
Time-course alterations of (a) the mean LIV, (b) the mean ${\mathrm{OCDS}}_{l}$ , (c) the spheroid volume, and the viable cell ratios based on (d) the ${\mathrm{3-dB}}^{2}$ LIV cut-off and (e) the 2 $\times 10^{-4}$ ${\mathrm{ms}}^{-1}$ ${\mathrm{OCDS}}_{l}$ cut-off.

Figure 5 summarizes the results of the quantitative analysis of the MCF-7 spheroid time course tissue viability alterations. The mean LIV [Fig. 5(a)], the mean ${\mathrm{OCDS}}_{l}$ [Fig. 5(b)], and the spheroid volume [Fig. 5(c)] are all plotted versus the measurement time points. The mean values of both the LIV and the ${\mathrm{OCDS}}_{l}$ are clearly decreasing over time. It should be mentioned that the mean ${\mathrm{OCDS}}_{l}$ [Fig. 5(b)] is lower than our previous study (Fig. 4(d) in [42]). This point is discussed in detail in Section 5.4. On the other hand, the spheroid volume is slightly increasing over time. Viable cell ratios based on the LIV and the ${\mathrm{OCDS}}_{l}$ cut-offs are also plotted versus time as shown in Figs. 5(d) and 5(e), respectively. These plots show that the viable cell ratio decreases over time.

A similar longitudinal measurement was performed on another MCF-7 spheroid. The protocol was identical to Study 1 except that the spheroid was seeded with 500 tumor cells whereas it was 1,000 cells for Study 1. Similar results to those in Figs. 3–5 were obtained (see Supplement 1).

### 4.2 Study 2: 3D drug response evaluation of the MCF-7 spheroid

#### 4.2.1 LIV imaging

**Fig. 6.**
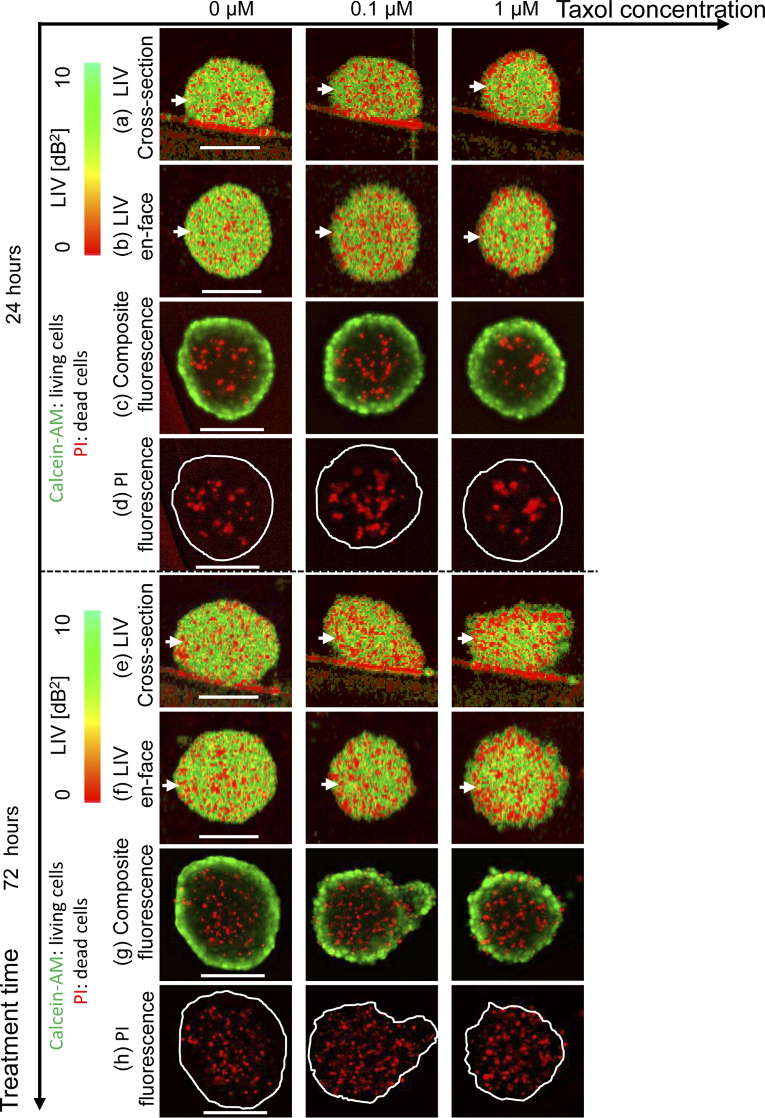
LIV imaging of MCF-7 spheroid treated with Taxol. (a)-(d) represent the cross-section, *en face*, composite fluorescence, an PI fluorescence images at the 24 h time point, respectively. The 72 h time point images [(e)-(h)] are displayed in the same manner. The scale bars represents 200 μ m in each case.

Figure 6 summarizes the LIV imaging results of an MCF-7 spheroid that was treated with Taxol for 24 and 72 h. The first to fourth rows [Figs. 6(a)–6(d)] show the images acquired at 24 h time point, including the fast-scan cross-section, the *en face* cross-section of the LIV, the composite fluorescence image of PI (red) and calcein-AM (green), and the single channel PI fluorescence image, respectively. The fifth to eighth rows [Figs. 6(e)–6(h)] represent the corresponding images acquired at the 72 h time point and are displayed in the same manner as Figs. 6(a)–6(d). In the control (0 μ M) case at the 24 h time point, almost the entire spheroid region shows a high LIV appearance, as shown in the cross-section [Fig. 6(a)] and the *en face* LIV images [Fig. 6(b)]. The composite fluorescence image presented in Fig. 6(c) shows living cells appearance (green fluorescence), which corresponds to the high LIV appearance. In contrast, the spheroid core exhibited a dead-cell fluorescence signal (red), as illustrated in Figs. 6(c) and 6(d). This is consistent with the low LIV (red) spots. Images with similar appearances were obtained in the control case at the 72 h time point [Figs. 6(e)–6(h)].

In the 0.1 μ M case (second column of Fig. 6), more low LIV (red) spots are visible than that were observed in the control case at the 24 h time point. The composite and PI fluorescence images show larger dead-cell (red) spots than that were shown in the control case, while the periphery shows the appearances of living cells (green). At the 72 h time point, the spheroid shows a non-spherical shape and almost the entire spheroid region shows a low LIV (red) signal. The fluorescence images show more dead-cell (red) spots than that were seen in the control case. These appearances may indicate the reduction of cell viability induced by application of the drug. It can thus be concluded that the LIV images are sensitive to tissue viability alterations induced even by a small drug concentration (0.1 μ M) and over a short treatment time (24 h).

When the drug concentration is increased further to 1 μ M (see the third column of Fig. 6), the spheroid periphery shows a clear low LIV signal (red) at the 24 h time point. However, the fluorescence images show similar appearances to those of the 0.1- μ M case. This low LIV signal covers almost all the spheroid region at the 72 h time point. The dead cells florescence (red) signal at 72 h time point spreads not only at the spheroid core but also at the periphery. Tissue necrotic cell death and tissue metabolic activity degradation in tumor spheroids, including MCF-7, are well known to be induced by the drug applications [7,14]. Therefore, the increase in the low LIV signal region caused by increasing both the drug concentration and the incubation time may indicate necrotic cell death and degradation of the tissue viability.

**Fig. 7.**
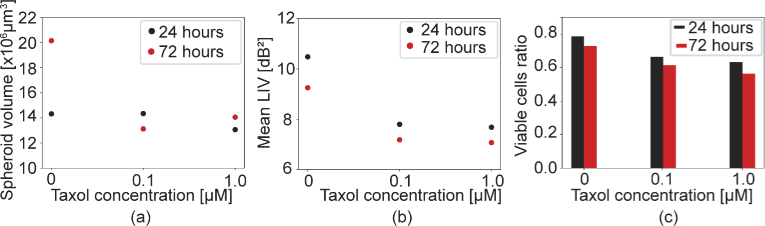
Quantitative analysis of the MCF-7 spheroid when treated with Taxol. (a) The spheroid volume, (b) the mean LIV, and (c) the viable cell ratio are plotted versus the drug concentration for the 24 h and 72 h time points.

Figure 7 summarizes the results of LIV-based quantitative analysis of the MCF-7 spheroid drug response. At the 24 h time point, the spheroid volumes in the 0 μ M and 0.1 μ M cases are almost identical, while that in the 1 μ M case shows a slightly smaller volume [Fig. 7(a)]. The dependence of the spheroid volume on the drug concentration is more pronounced at the 72 h time point. The mean LIV [Fig. 7(b)] of the 0.1 μ M case is evidently smaller than that in the control case, while the mean LIVs of 0.1 μ M and 1.0 μ M are almost identical. Figure 7(c) shows that smaller viable cell ratios are found with higher drug concentrations at both of the time points.

#### 4.2.2 ${\mathrm{OCDS}}_{l}$ imaging

**Fig. 8.**
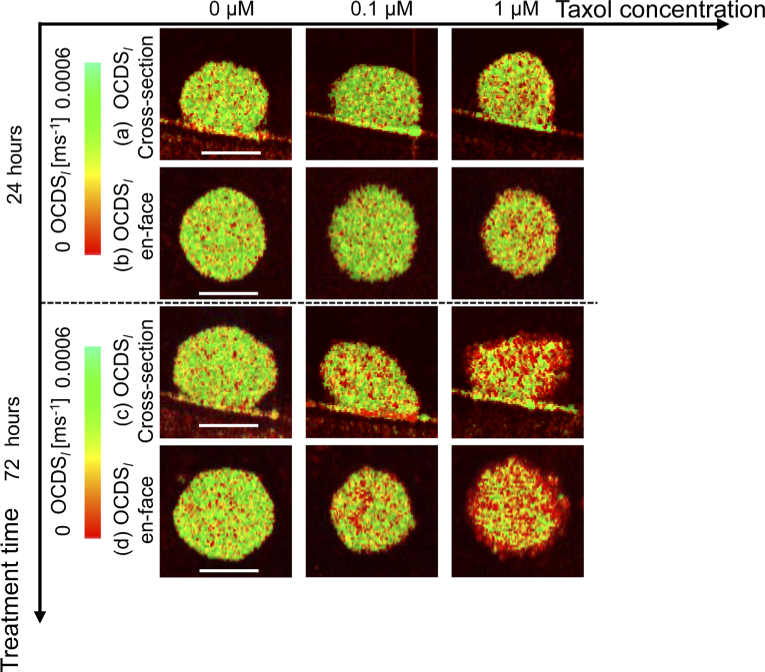
${\mathrm{OCDS}}_{l}$ imaging of the MCF-7 spheroid when treated with Taxol. (a) and (b) respectively represent the cross-section and *en face* ${\mathrm{OCDS}}_{l}$ images at the 24 h time point, while the corresponding 72 h time point images are shown in (c) and (d), respectively. The scale bars represent 200 μ m.

Figure 8 summarizes the ${\mathrm{OCDS}}_{l}$ imaging results for the same MCF-7 spheroid drug response study presented in Section 4.2.1. At the 24 h time point, the cross-sectional and *en face* ${\mathrm{OCDS}}_{l}$ results [Figs. 8(a) and (b)] at a concentration of 0 μ M show high ${\mathrm{OCDS}}_{l}$ over almost the entire spheroid region. A similar appearance was observed after increasing the drug concentration to 0.1 μ M. In contrast, the 1 μ M case shows more low ${\mathrm{OCDS}}_{l}$ (red) signals, however it is not as evident as that was observed in LIV images [Fig. 6].

In the 72 h case, more low ${\mathrm{OCDS}}_{l}$ (red) signals are observed at higher drug concentrations. These results are consistent with the LIV results presented in Fig. 6.

The results also suggest that the ${\mathrm{OCDS}}_{l}$ is more sensitive to the late-stage drug-induced alteration of the spheroid’s viability than to mild-stage alteration.

**Fig. 9.**
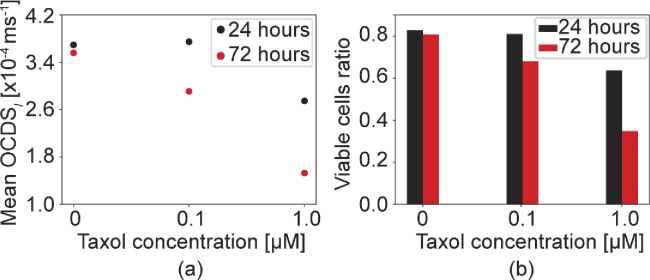
${\mathrm{OCDS}}_{l}$ based quantitative analysis results for the same MCF-7 spheroid that was shown in Fig. 7. The (a) mean ${\mathrm{OCDS}}_{l}$ and (b) viable cell ratios are plotted versus the drug concentrations.

The quantitative analysis results of the MCF-7 drug response as measured via the ${\mathrm{OCDS}}_{l}$ method are presented in Fig. 9. At 24 h time point, the mean ${\mathrm{OCDS}}_{l}$ [Fig. 9(a)] does not show a clear difference between the 0 μ M and 0.1 μ M cases, while the 1 μ M case shows an evidently lower ${\mathrm{OCDS}}_{l}$ than the other two cases. In contrast, at the 72 h time point, the mean ${\mathrm{OCDS}}_{l}$ decreases linearly as the drug concentration increases. At the 24 h time point, the viable cell ratios are almost the same for the 0 μ M and 0.1 μ M cases, while the ratio is low for 1 μ M. In contrast, at the 72 h time point, the viable cell ratio decreases linearly as the drug concentration increases, as shown in Fig. 9(b).

## 5. Discussion

### 5.1 Dynamics Imaging reveals tissue necrosis and metabolic activity

In this study, we have presented a 3D OCT-based method for the visualization and quantification of tissue dynamics, which are mainly related to the sub-cellular motion. The alterations in the magnitude and speed of this sub-cellular motion may be closely related to the physiological properties of the tissue, e.g., the tissue’s metabolic activity. The tissue’s metabolic activity is known to be a series of chemical reactions that occur in living organisms to maintain and run the cellular processes [49] along with tissue abnormalities such as apoptosis, necrosis, and oncosis [50–52].

Large cultured tumor spheroids are known to have a necrotic core and a viable outer layer [5,53,54]. The viable cells at the periphery of the spheroid have an intact nuclei and cell membranes and this is associated with both rapid proliferation and high metabolic activity, while the necrotic core consists of cells that have ruptured nuclei and membranes [55]. These necrotic cells lose their activity because they are deprived from nutrients and because of the accumulation of toxic wastes [55–57].

The dynamics OCT methods showed that the spheroid core exhibited low LIV and ${\mathrm{OCDS}}_{l}$ , as illustrated at the 0 h time point of study-1 (Section 4.1), which could be an indicator of the necrosis occurring at the spheroid core. In contrast, the spheroid’s outer layer shows high LIV and ${\mathrm{OCDS}}_{l}$ . These high dynamics may be accounted for by the high metabolic activity at the proliferative spheroid rim.

Necrotic cell death and tissue metabolic activity degradation in tumor spheroids, including the MCF-7 spheroid, is well known to be induced by the drug applications [7,14]. Therefore, the reduced LIV and ${\mathrm{OCDS}}_{l}$ in response to the increased drug concentration presented in Section 4.2 could be related to the drug-induced necrotic cell death and metabolic activity degradation, which indicates the potential capability of our 3D dynamics OCT method in drug screening application.

### 5.2 Comparison of tissue dynamics contrast with bright field and staining fluorescence imaging

#### 5.2.1 General comparison

Fluorescence microscopic imaging using suitable staining solutions is a gold standard method for tissue viability imaging applications, including tumor spheroid imaging. Bright field microscopy is also used in tumor spheroid evaluation. Although these methods represent current gold standards, the 3D dynamics imaging method presented here offers several advantages. First, the proposed method is non-destructive and label-free, i.e., it is non-invasive. Second, the method provides a 3D tissue viability contrast. Third, it enables full-depth imaging of the thick cultivated tissues such as the tumor spheroid, while the conventional microscopes have only limited penetration depths. Fourth, the LIV and ${\mathrm{OCDS}}_{l}$ give absolute values, so they are readily quantified as presented in Section 4. In contrast, fluorescence signals are not really quantitative. The strength of the fluorescence signal is heavily affected by several factors including imaging conditions, superior tissues, and sample morphology. And hence, the fluorescence signal strengths are hard to be compared among the images and even within a single image.

*En face* imaging, which became available through the volumetric dynamics OCT imaging technique, is another important feature of the proposed method. This approach is particularly useful to compare the dynamics OCT imaging results with those of the conventional microscopic imaging modalities, such as fluorescence and bright field microscopes, which are *en face* modalities.

#### 5.2.2 *En face* image similarity

A large-sized spheroid was grown by initially seeding 5,000 MCF-7 cells using the same seeding protocol that was explained in Section 3.1. After cultivation for 6 days, the spheroid was measured using a bright field microscope (IX71, Olympus) with an objective lens with 4 × magnification and NA of 0.13, a fluorescence microscope, and OCT.

**Fig. 10.**
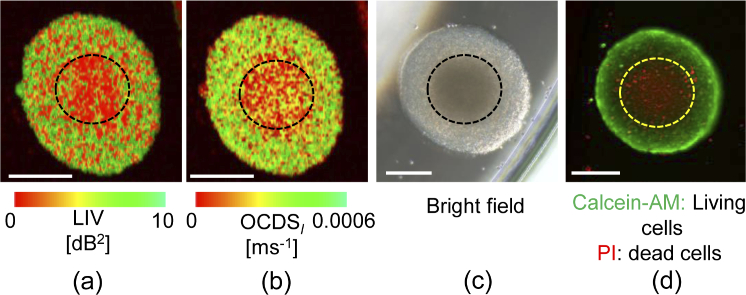
*En face* imaging comparison between (a) LIV, (b) ${\mathrm{OCDS}}_{l}$ , (c) bright field, and (d) fluorescence microscope techniques. The scale bars represent 200 μ m.

Figure 10 shows a comparison between the *en face* microscopic images acquired using the (a) LIV, (b) ${\mathrm{OCDS}}_{l}$ , (c) the bright field, and (d) fluorescence technique. The LIV image in Fig. 10(a) shows a clear low LIV (necrotic) signal at the spheroid core, as highlighted by the black dashed circle, while a high LIV (metabolic active viable cells) signature is shown at the periphery region. The same appearance is obtained by the ${\mathrm{OCDS}}_{l}$ imaging as illustrated in Fig. 10(b). The contrast between the core and the outer shell is observed more clearly with ${\mathrm{OCDS}}_{l}$ than with the LIV method. This difference occurs because the LIV method is sensitive only to the magnitude of the sub-cellular motion, while the ${\mathrm{OCDS}}_{l}$ is selectively sensitive to the slow sub-cellular motion.

The bright field image presented in Fig. 10(c) shows that the spheroid’s core region exhibits a dark appearance, as highlighted by the black dashed circle, which corresponds to the spheroid core necrosis. In contrast, the spheroid periphery shows a bright appearance, which indicates that the cells remain viable at the spheroid periphery. This appearance is consistent with those obtained using the LIV and ${\mathrm{OCDS}}_{l}$ techniques.

The fluorescence microscopic image presented in Fig. 10(d) shows that the spheroid core exhibited a red fluorescence (necrotic dead cells indicator), as highlighted by the yellow dashed circle. In contrast, the spheroid periphery showed green fluorescence (viable cells indicator). These appearances are also consistent with those obtained from the LIV and ${\mathrm{OCDS}}_{l}$ approaches.

All of these methods visualized the tumor spheroid’s necrotic core and viable outer rim, but among them, only the dynamics OCT method is completely label-free, non-invasive, and provides 3D imaging.

#### 5.2.3 Mimicking fluorescence imaging by OCT

Label-free tissue specific contrast can be also obtained via application of appropriate thresholds to the LIV and ${\mathrm{OCDS}}_{l}$ images, with values of 3 ${\mathrm{dB}}^{2}$ and 2 $\times 10^{-4}$ ${\mathrm{ms}}^{-1}$ , respectively. The tissue regions with higher tissue dynamics than the threshold values can be treated as viable cells regions, while those with values that are equal to or lower than the threshold can be treated as necrotic tissue regions.

**Fig. 11.**
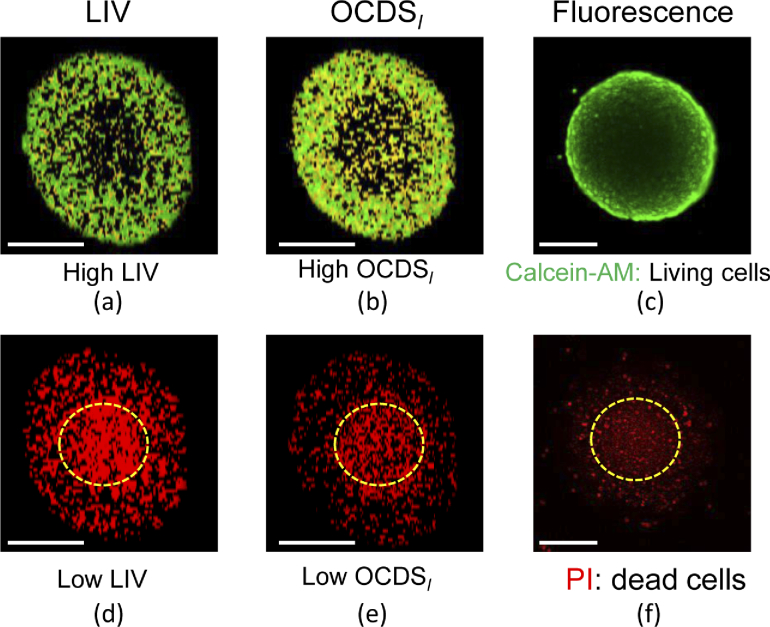
Fluorescence imaging mimicking by dynamics OCT. The first row represents the imaging comparison between (a) LIV based viable cells, (b) ${\mathrm{OCDS}}_{l}$ based viable cells, and (c) calcein-AM; living cells fluorescence, while the second row represents the comparison between (d) LIV based necrotic cells, (e) ${\mathrm{OCDS}}_{l}$ based necrotic cells, and (f) PI; dead cells fluorescence. The scale bars represent 200 μ m.

Figure 11 shows an *en face* comparison between the results obtained from the dynamics imaging (LIV and ${\mathrm{OCDS}}_{l}$ ) techniques and the separated fluorescence channels (green for living cells and red for dead cells). As Fig. 11(a) and (b) show, only the spheroid periphery shows high LIV and high ${\mathrm{OCDS}}_{l}$ . This appearance is similar to that of the calcein-AM staining fluorescence microscopic image shown in Fig. 11(c). Contrastingly, Fig. 11(d) and (e) show that the spheroid core exhibited red (low dynamics) appearances, as highlighted by the yellow dashed circles, where the more central region shows the more intense appearance of red. In addition, some regions in the spheroid periphery also show a low dynamics appearance. This appearance is consistent with that obtained from the PI staining fluorescence method shown in Fig. 11(f). Therefore, we can conclude that the dynamics imaging technique provides images with similar appearances to those from fluorescence microscopy, but in a completely label-free manner.

### 5.3 Possible virtual histology via multi-contrast OCT imaging

Two volumetric tissue dynamics contrast methods have been presented, comprising the LIV and ${\mathrm{OCDS}}_{l}$ methods. These two quantities are sensitive to different aspects of the tissue’s activity. The LIV method is sensitive to the magnitude of the temporal fluctuations of the OCT signal, while the ${\mathrm{OCDS}}_{l}$ is sensitive to the slow tissue dynamics. In addition, the OCT used in this study is a Jones matrix-based polarization-sensitive OCT, which also provides birefringence and a degree of polarization uniformity. Further more, this also allows an attenuation coefficient image to be obtained.

These multiple contrasts can be used to create new sets of contrasts by, for example, principal component analysis [58–62], machine learning-based tissue segmentation [63,64], and multi-contrast-based tissue segmentation [23,65,66]. In future work, these methods will provide an OCT-based histopathology-like contrast.

### 5.4 Benefits and limitations of the 3D dynamics imaging method over previous 2D method

The presented 3D dynamics scanning protocol has several benefits and some limitations over our previous 2D protocol [42]. At first, this scanning protocol allows volumetric evaluation of samples in less than one minute. This volumetric capability allowed the quantitative evaluations of the entire spheroid, such as volume analysis, volumetric mean LIV, ${\mathrm{OCDS}}_{l}$ , and viable cell ratio analyses (Sections 4.1 and 4.2). In addition, it provides the *en face* dynamics OCT images, which can be directly compared to the *en face* fluorescence images as presented in Sections 4.2 and 5.2.

However, the 3D dynamics scanning protocol also has some drawbacks. For example, the time separation between adjacent frames is 204.8 ms, which is significantly larger than 12.8 ms of our previous 2D protocol [42]. It has prevented using the same correlation delay range for ${\mathrm{OCDS}}_{l}$ computation with our previous protocol. It was [64.0, 627.2 ms] for the previous 2D protocol and [204.8, 1228.8 ms] for the current 3D protocol. It may account for the different mean ${\mathrm{OCDS}}_{l}$ values of the present study and the previous study [42].

In addition, the ${\mathrm{OCDS}}_{e}$ (early OCDS) presented in the previous study cannot be obtained with the present 3D protocol. The ${\mathrm{OCDS}}_{e}$ captures fast tissue dynamics occurring in a few tens of milliseconds [42], and its correlation decay range is [12.8, 64.0 ms]. The maximum of this range is still smaller than the frame separation time of the present 3D protocol. Further improvement of the scanning protocol may overcome these issues [67].

## 6. Conclusion

A 3D tissue dynamics imaging method has been introduced in this work and its utility for the evaluation of tumor spheroids has been investigated. The time-course tissue viability alterations and drug responses of MCF-7 tumor spheroids have been visualized via 3D tomography. A quantification of the tumor spheroid’s tissue activity was also presented. *En face* comparison between the gold standard fluorescence microscopy method and our tissue dynamics imaging technique showed a good consistency.

These results suggest that the presented 3D OCT-based tissue dynamics imaging method could become a useful tool for quality control and drug response evaluation of tumor spheroids.

## Data Availability

Data underlying the results presented in this paper are not publicly available at this time but may be obtained from the authors upon reasonable request.

## Supplemental document

See Supplement 1 for supporting content.
