# Supplementary material for: Three-dimensional dynamics optical coherence tomography for tumor spheroid evaluation
Source: Biomed Opt Express. 2021 Oct 12;12(11):6844–63. doi: 10.1364/BOE.440444 (PMC8606131; doi:10.1364/BOE.440444)
Supplement: Supplementary file 1 [file boe-12-11-6844-s001.pdf]

Supplemental Document

Biomedical Optics EXPRESS

Three-dimensional dynamics optical coherence tomography for tumor spheroid evaluation: supplement

IBRAHIM ABD EL-SADEK,[1,2] ARATA MIYAZAWA,[3] LARINA TZU-WEI SHEN,[4] SHUICHI MAKITA,[1] PRADIPTA MUKHERJEE,[1] ANTONIA LICHTENEGGER,[1,5] SATOSHI MATSUSAKA,[4] AND YOSHIAKI YASUNO[1,*]

[1] *Computational Optics Group, University of Tsukuba, Tsukuba, Ibaraki 305-8573, Japan*
[2] *Department of Physics, Faculty of Science, Damietta University, New Damietta City, 34517, Damietta, Egypt*
[3] *Sky Technology Inc., Tsukuba, Ibaraki 305-0032, Japan*
[4] *Clinical Research and Regional Innovation, Faculty of Medicine, University of Tsukuba, Ibaraki 305-8575, Japan*
[5] *Center for Medical Physics and Biomedical Engineering, Medical University of Vienna, Währinger Gürtel 18-20, 4L, 1090, Vienna, Austria*
[*] *yasuno@optlab2.bk.tsukuba.ac.jp*

This supplement published with Optica Publishing Group on 12 October 2021 by The Authors under the terms of the Creative Commons Attribution 4.0 License in the format provided by the authors and unedited. Further distribution of this work must maintain attribution to the author(s) and the published article's title, journal citation, and DOI.

Supplement DOI: https://doi.org/10.6084/m9.figshare.16708702

Parent Article DOI: https://doi.org/10.1364/BOE.440444

Three-dimensional dynamics optical coherence tomography for tumor spheroid evaluation

Here we present an additional time-course evaluation of Human breast adenocarcinoma (MCF-7) spheroid formed by initially seeding 500 tumor cells. The results are consistent with the same cell line presented in the full length manuscript.

1. RESULTS

Figure S1 summarizes the LIV time course of MCF-7 spheroid formed by initially seeded with 500 human breast cancer cells. At 0 h, the spheroid shows high LIV layer surrounding the low LIV region as shown in the volume rendering and cross-sections. By following the time course, the high LIV at the spheroid periphery is fading over the time until the low LIV signal (red) covers almost all the spheroid volume. These results are consistent with Fig. 3 in the full length manuscript.

Figure S2 shows the OCDS_l time course of the same spheroid presented in Fig. S1. And the images show similar tendencies as observed by LIV. The spheroid core shows low OCDS_l, while the periphery shows high OCDS_l at 0-h time point and this high OCDS_l is fading over time. These results are also consistent with that presented in Fig. 4 in the manuscript.

Figure S3 summarizes the quantitative analysis of the time course spheroid data presented in Figs. S1 and S2. The plots show similar tendencies to that presented in Fig. 5 in the manuscript. Namely, clear reduction of the mean LIV [Fig. S3(a)], OCDS_l [Fig. S3(b)], LIV cut-off based viable cell ratio [Fig. S3(d)], and that based on OCDS_l cut-off [Fig. S3(e)] were found over time. In contrast, the spheroid volume is slightly increasing along the time as shown in Fig. S3(c).

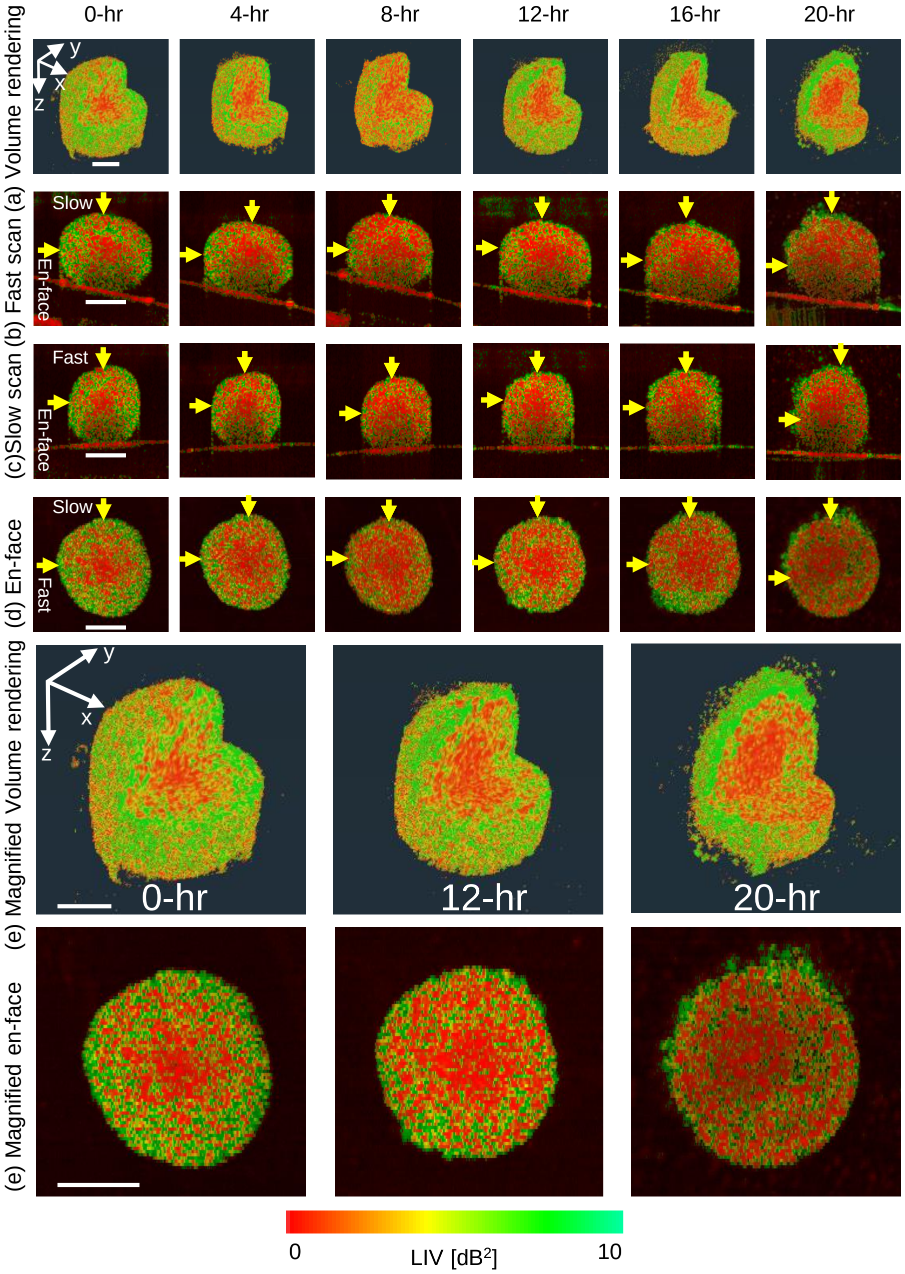

Fig. S1. Time-course LIV visualization of MCF-7 spheroid formed by initial seeding of 500 tumor cells. The first to fourth rows show time-course images of (a) the cut-away volume rendering, (b) the cross-section along the fast scan direction, (c) the cross-section along the slow scan direction, and (d) the *en face* cross-section, respectively. The fifth and sixth rows show magnified images of (e) the volume rendering and (f) the *en face* cross-section, respectively. All the scale bars represent 200 μm.

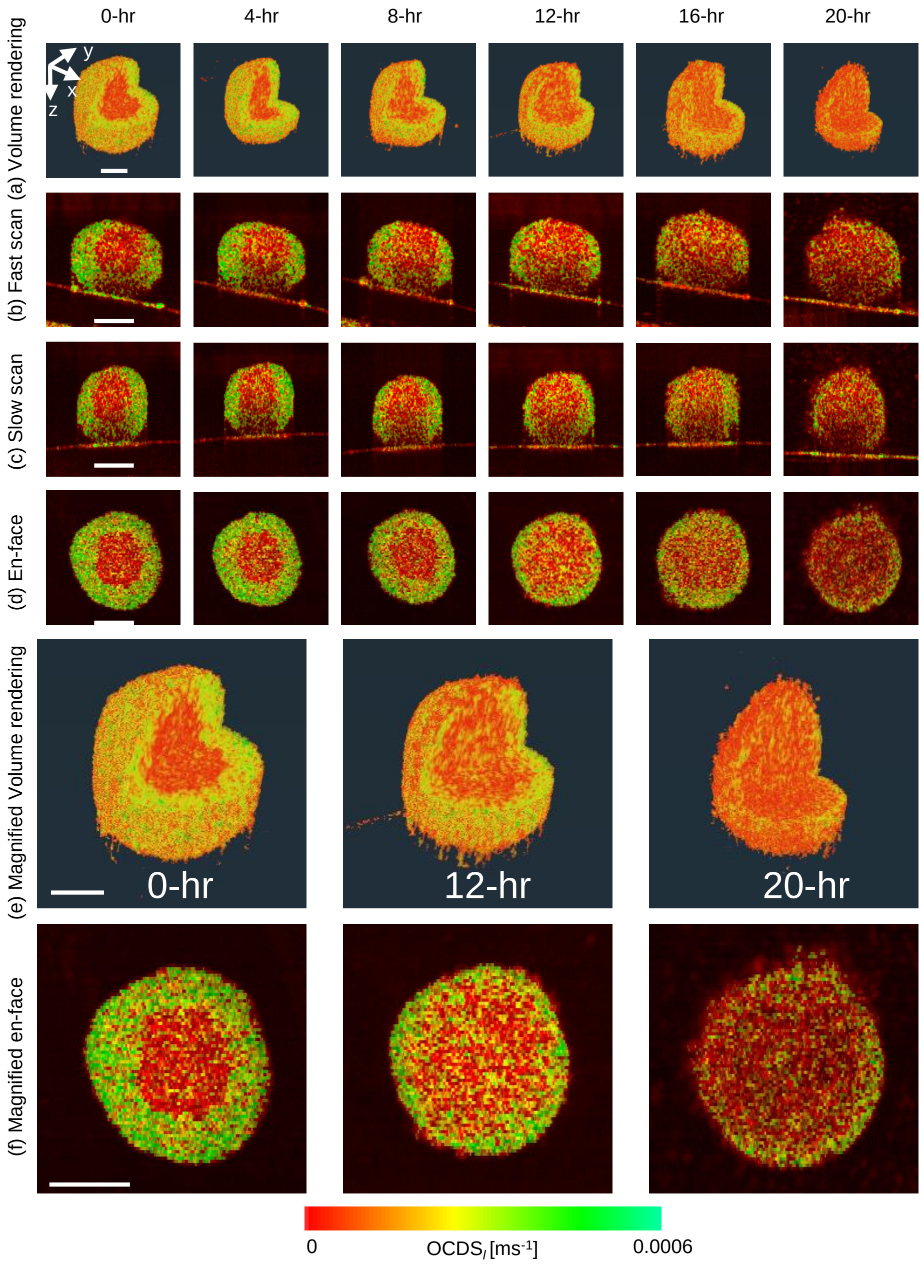

Fig. S2. Time-course OCDS$_l$ of MCF-7 spheroid formed by initially seeding of 500 tumor cells. The images are displayed in the same manner as those shown in Fig. S1.

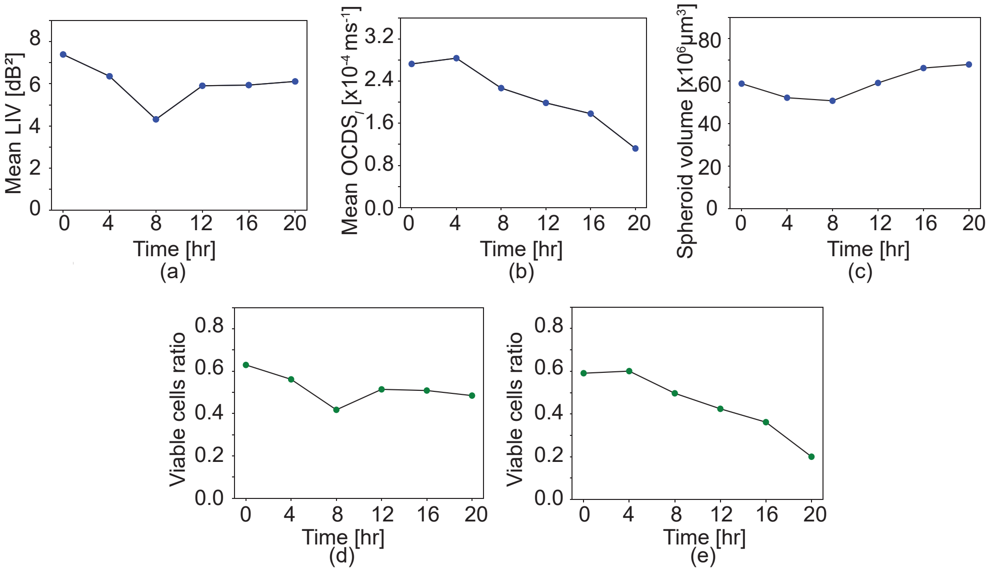

Fig. S3. Time-course alterations of (a) the mean LIV, (b) the mean OCDS$_l$, (c) the spheroid volume, and the viable cell ratios based on (d) the 3-dB^2 LIV cut-off and (e) the 2 $\times 10^{-4}$ ms^{-1} OCDS$_l$ cut-off.
